# External Carotid Artery Position Relative to the Greater Horn of the Hyoid: A Comprehensive Bilateral Analysis of Anatomical Variations

**DOI:** 10.7759/cureus.101903

**Published:** 2026-01-20

**Authors:** Viviana Dincă, Rodica N Calotă, Marius I Rusu, Răzvan C Tudose, Cătălin C Dumitru, Mugurel C Rusu

**Affiliations:** 1 Anatomy, Faculty of Dentistry, Carol Davila University of Medicine and Pharmacy, Bucharest, ROU; 2 Economic Informatics, Faculty of Cybernetics, Statistics and Economic Informatics, Bucharest University of Economic Studies, Bucharest, ROU; 3 Research, Dr. Carol Davila University Emergency Central Military Hospital, Bucharest, ROU

**Keywords:** anatomical variation, bilateral asymmetry, computed tomography angiography, external carotid artery, hyoid bone

## Abstract

Purpose

The external carotid artery (ECA) exhibits variable relationships with the greater horn of the hyoid bone (GHHB), which is relevant to cervical surgery. We aimed to classify ECA positions relative to the GHHB, describe bilateral patterns, and provide prevalence data for surgical planning.

Methods

A retrospective cross-sectional CTA study was conducted in 115 adults (75 males and 40 females), with a mean age of 58.3 ± 14.2 years. Eleven ECA-GHHB positional types were defined. Positions were recorded bilaterally, combined into symmetry/asymmetry patterns, and analysed with descriptive statistics, Chi-square tests, McNemar tests, binomial tests, and logistic regression (EViews 13.0, Quantitative Micro Software, Irvine, CA, USA).

Results

Eleven ECA positions were identified. Lateral (34.8%) and postero-lateral (32.6%) positions dominated (67.4% combined). Overall, 32 bilateral combinations were recorded. Lateral position showed a strong right-side preference (46.1% right vs 23.5% left; p < 0.0001). Bilateral asymmetry was more frequent than symmetry (58.3% vs 41.7%; p = 0.093). Lateralised ECAs (lateral to the internal carotid artery (ICA)) occurred in 3.5% of sides, mainly on the right, and could not be related to the GHHB. No significant gender differences were found (p > 0.05).

Conclusion

ECA-GHHB relationships are highly variable, with dominant lateral/postero-lateral patterns, a marked right-sided lateral preference, and frequent bilateral asymmetry. The proposed classification and prevalence data support preoperative risk assessment and highlight the need for patient-specific imaging in cervical surgery.

## Introduction

The carotid system supplies the head and neck via the common carotid arteries (CCAs), which bifurcate into the external carotid artery (ECA) and internal carotid artery (ICA) at about the upper border of the thyroid cartilage (C3-C4) [1-4]. The right CCA arises from the brachiocephalic trunk, whereas the left CCA arises from the aortic arch. The ECA usually lies antero-medial to the ICA and gives eight named branches: superior thyroid, lingual, facial, ascending pharyngeal, occipital, posterior auricular, maxillary, and superficial temporal arteries [5-7]. In the lateralised ECA variant, the ECA runs lateral or postero-lateral to the ICA, and its anterior branches cross the ICA before assuming their usual course [8].

The carotid arteries and the hyoid bone exhibit substantial interindividual variation [2,3]. These relationships matter in carotid surgery, neck dissection, thyroid surgery, and in interpreting imaging [2,9-16]. Aberrant topography may predispose to compression, stenosis, pseudoaneurysm, stroke, or cranial nerve palsy [9,11-18].

Existing work often focuses on the carotid bifurcation (CB) level or global carotid-hyoid topography rather than ECA-specific relations to the greater horn of the hyoid bone (GHHB) [1-3,10]. Data on bilateral patterns and detailed ECA positional variants remain scarce.

We therefore aimed to: (1) define a reproducible classification of ECA positions relative to the GHHB on CTA; (2) describe bilateral combinations and symmetry/asymmetry; (3) quantify the prevalence of lateralised ECAs; and (4) explore side- and gender-related associations. The goal is to provide concise, imaging-based anatomical data directly usable in surgical planning.

## Materials and methods

Study design and population

A retrospective cross-sectional CTA study was conducted in line with the Declaration of Helsinki and approved by the Institutional Ethics Committee (no. 737; dated November 1, 2024). Given the anonymised retrospective nature of the data, informed consent was waived.

We analysed 115 adult CTA examinations (75 males, 40 females; mean age 58.3 ± 14.2 years; range 18-85 years). Inclusion criteria were age ≥18 years, bilateral coverage from the aortic arch to the skull base, adequate image quality, clearly visible CCAs, CBs, ECAs, ICAs, and GHHBs bilaterally, and no motion or contrast artefacts affecting the region of interest. Exclusion criteria included cervical surgery or trauma affecting carotid-hyoid anatomy, congenital cervical or hyoid anomalies, tumoural or inflammatory processes involving the carotids or hyoid, severe atherosclerosis (>50% stenosis), incomplete or suboptimal contrast, significant motion artefacts, or previous neck irradiation.

Imaging protocol

CTA was performed on a 64-slice scanner (SOMATOM Definition AS+, Siemens Healthcare, Erlangen, Germany). Typical parameters were 120 kVp, 200-300 mAs (automatic modulation), slice thickness 0.75 mm, reconstruction increment 0.5 mm, pitch 1.2, rotation time 0.6 s, and field of view 25 cm. Contrast consisted of 80-100 mL of iodinated contrast (Ultravist 370) at 4-5 mL/s, followed by 50 mL saline; bolus tracking was performed in the ascending aorta with a three-second delay after 100 HU. Data were reconstructed with a soft-tissue kernel (512 × 512 matrix). Axial, coronal, and sagittal multiplanar reconstructions (MPRs) at 1-mm thickness, and 3D volume-rendered images (Horos v4.0, Purview, Annapolis, MD, USA) were used for assessment.

Positional classification

**Figure 1:**
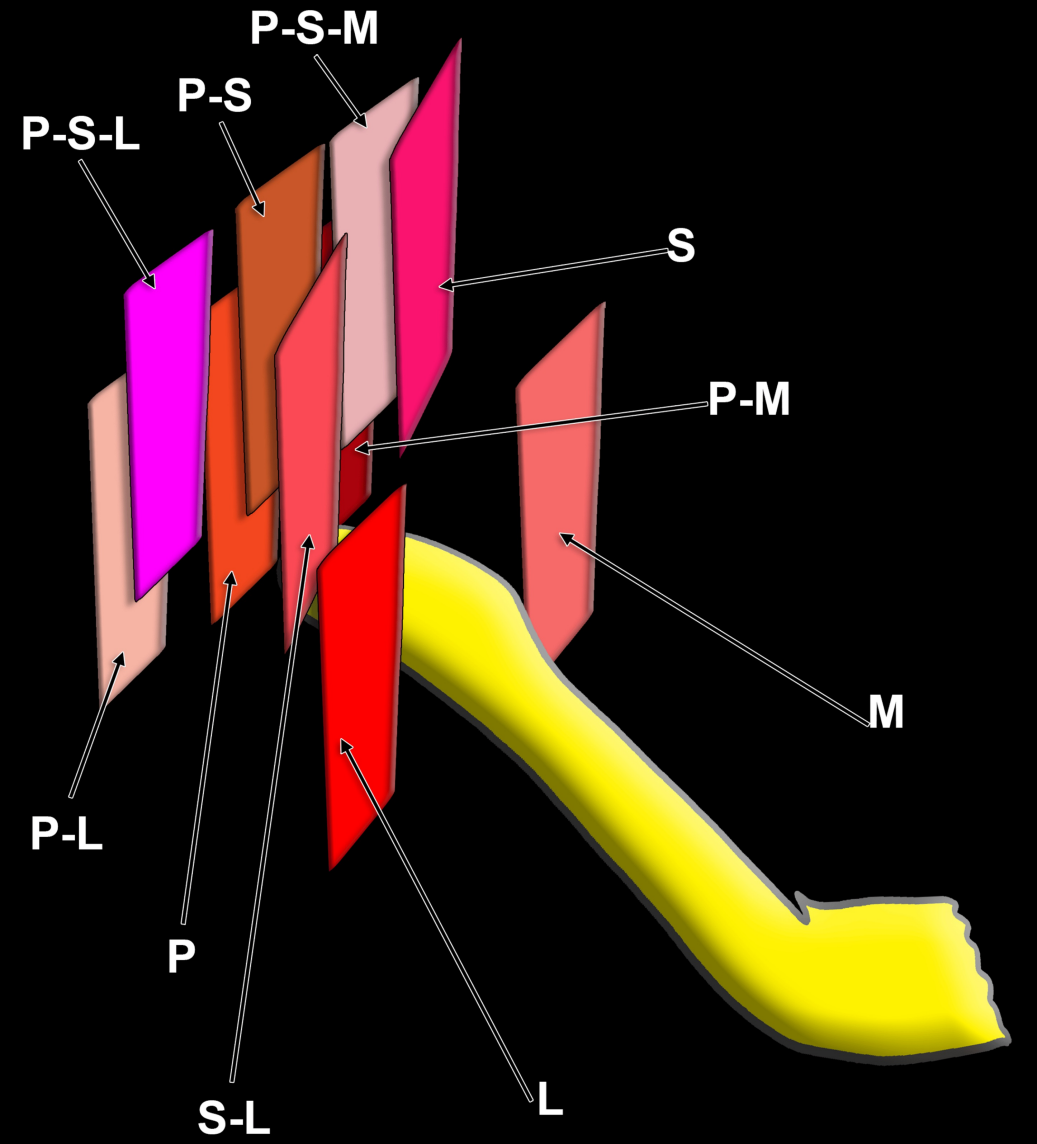
Positional types of the external carotid artery, as related to the greater hyoid horn. M: medial; L: lateral; S-L: supero-lateral; P-L: postero-lateral; P-S-L: postero-supero-lateral; P-S: postero-superior; P-S-M: postero-supero-medial; S: superior; P-M: postero-medial

We identified the CCA, CB, ECA, ICA, and GHHB on both sides. Based on anatomical observations and location patterns, we defined 11 ECA-GHHB positional types (Figure 1). These included lateral (ECA lateral to GHHB), medial (ECA medial to GHHB), posterior (ECA posterior to GHHB), postero-lateral (ECA posterolateral to GHHB), postero-medial (ECA posteromedial to GHHB), postero-superior (ECA posterosuperior to GHHB), postero-supero-lateral (ECA posterosuperolateral to GHHB), postero-supero-medial (ECA posterosuperomedial to GHHB), superior (ECA superior to GHHB), and supero-lateral (ECA superolateral to GHHB). The 11th type was the lateralised ECA, in which the ECA lies lateral to the ICA within the carotid triangle, representing a true lateralised variant; in these cases, the ECA position could not be assigned relative to the GHHB.

For each subject, the right and left ECA positions, bilateral combination, and symmetry (identical positions) versus asymmetry (different positions) were recorded.

Observer protocol and reliability

All authors independently reviewed all CTA studies. Disagreements were resolved by consensus. A pilot analysis of 20 random cases was used to evaluate inter-observer reliability (Cohen’s kappa). Agreement was substantial (κ = 0.82; 95% CI: 0.71-0.93). Intra-observer reliability was assessed in 25 randomly selected cases, reassessed after four weeks by the same observer (κ = 0.91; 95% CI: 0.84-0.98).

Data management

Age, gender, and ECA positional data were entered in a standardised spreadsheet with predefined fields and validation rules. Each case received a unique anonymised ID. Bilateral positional combinations were coded as ordered pairs (right/left). Symmetry was defined as the condition of identical positions on both sides.

Statistical analysis

Analyses were performed in EViews 13.0 (Quantitative Micro Software, Irvine, CA, USA). Variables were binary (gender and symmetry) or categorical (11 ECA positions). We used descriptive statistics (frequencies and percentages); cross-tabulations of right/left positions and bilateral combinations; binomial tests (symmetry versus asymmetry against 50:50); Chi-square tests of independence (gender versus ECA positions); McNemar tests (paired right versus left side comparisons); Chi-square goodness-of-fit tests (uniform versus observed positional distribution); and binary logistic regression (association of specific ECA positions with gender and side). Assumptions (independence and expected cell counts ≥5) were checked, and no missing data were present. Statistical significance was set at α = 0.05. No correction for multiple testing was applied, given the exploratory design; effect sizes and confidence intervals were used to aid interpretation.

## Results

Sample characteristics

We analysed 115 subjects, including 75 males (65.2%) and 40 females (34.8%), with a male-to-female ratio of 1.88:1. The mean age was 58.3 ± 14.2 years (range 18-85 years). All subjects met the inclusion criteria and showed bilateral coverage of the cervical vascular anatomy from the aortic arch to the skull base. Image quality was adequate in all cases, with clear visualisation of the CCA, CB, ECA, ICA, and GHHB bilaterally.

Unilateral ECA-GHHB positions

Across 230 sides, 11 positional types were identified (Table 1). Lateral and postero-lateral positions predominated.

**Table 1:** Position of the external carotid artery (ECA) relative to the greater hyoid horn (GHHB), by side and combined (N = 230 sides). Data are presented as absolute counts (N) and percentages (%), calculated per side. Statistical significance was defined as p < 0.05. The distribution of ECA positions was tested using a Chi-square goodness-of-fit test (χ² = 389.157, p < 0.001). Lateralised ECAs are listed separately.

ECA-Hyoid Position	Right Side (N = 115)	Left Side (N = 115)	Overall Sample (N = 230 sides)
Lateral	53 (46.1%)	27 (23.5%)	80 (34.7%)
Postero-lateral	34 (29.6%)	41 (35.7%)	75 (32.6%)
Postero-supero-lateral	7 (6.1%)	12 (10.4%)	19 (8.3%)
Supero-lateral	7 (6.1%)	7 (6.1%)	14 (6.1%)
Postero-medial	2 (1.7%)	5 (4.3%)	7 (3.0%)
Medial	1 (0.9%)	4 (3.5%)	5 (2.2%)
Posterior	1 (0.9%)	13 (11.3%)	14 (6.1%)
Postero-superior	1 (0.9%)	4 (3.5%)	5 (2.2%)
Postero-supero-medial	1 (0.9%)	0 (0.0%)	1 (0.4%)
Superior	1 (0.9%)	1 (0.9%)	2 (0.9%)
Lateralised ECA	7 (6.1%)	1 (0.9%)	8 (3.5%)

**Figure 2:**
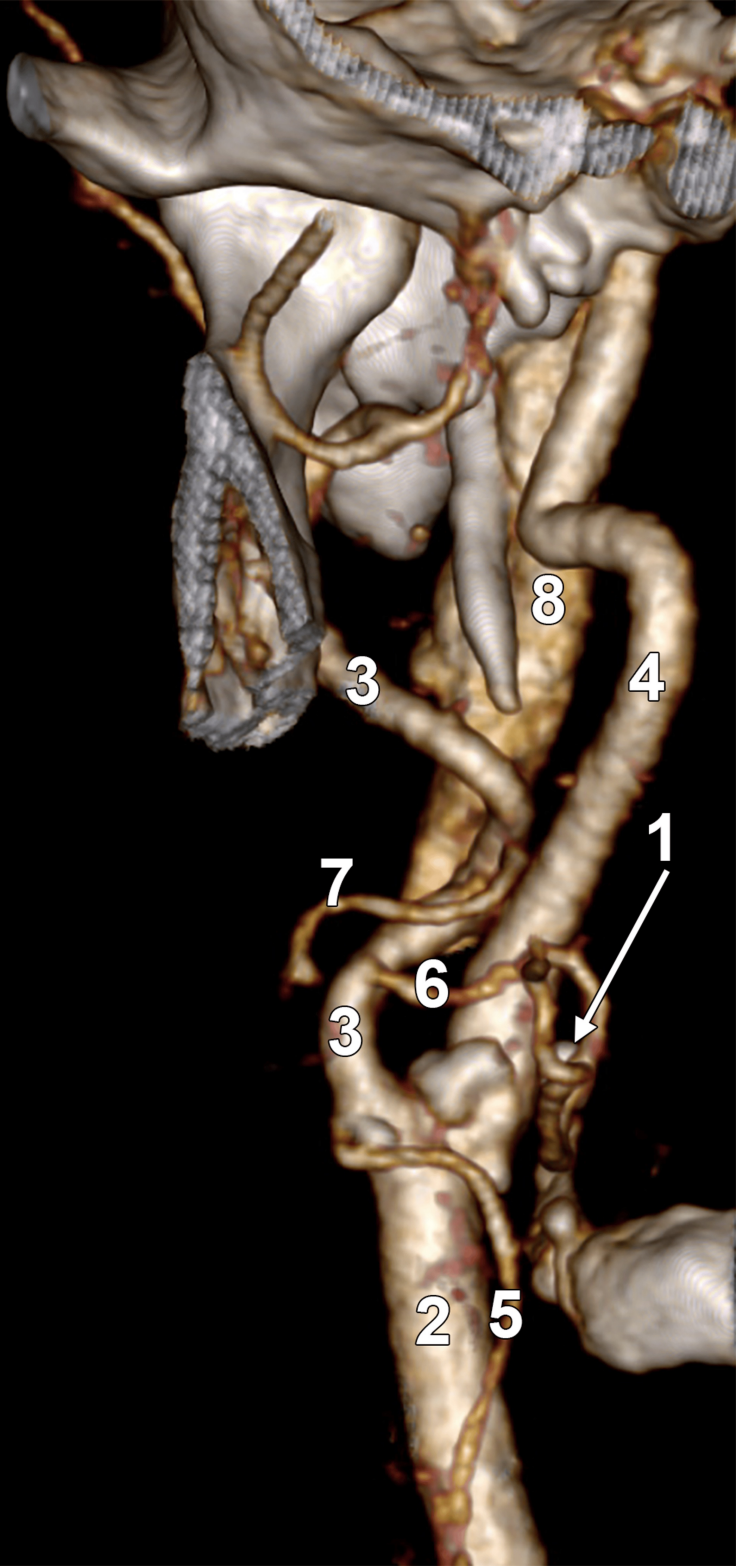
Lateralised right external carotid artery. Three-dimensional volume rendering, anterior view. 1, greater hyoid horn; 2, common carotid artery; 3, external carotid artery; 4, internal carotid artery; 5, superior thyroid artery; 6, lingual artery; 7, facial artery; 8, internal jugular vein.

In the 115 subjects, lateralised ECAs were seen in seven right (6.1%) and one left (0.9%) carotid triangle (Figure 2). No bilateral lateralised ECAs were found; thus, 8 of 230 sides (3.5%) showed this variant.

Bilateral combinations and symmetry

Overall, 32 bilateral combinations of ECA positions were documented. The most frequent patterns in the total sample were lateral/lateral (22 cases, 19.1%), lateral/postero-lateral (19 cases, 16.5%), and postero-lateral/postero-lateral (17 cases, 14.8%).

Other recurrent combinations included postero-lateral/posterior (seven cases; 6.1%), lateral/supero-lateral (four cases; 3.5%), postero-lateral/lateral (four cases; 3.5%), and postero-supero-lateral/postero-supero-lateral (four cases; 3.5%).

**Figure 3:**
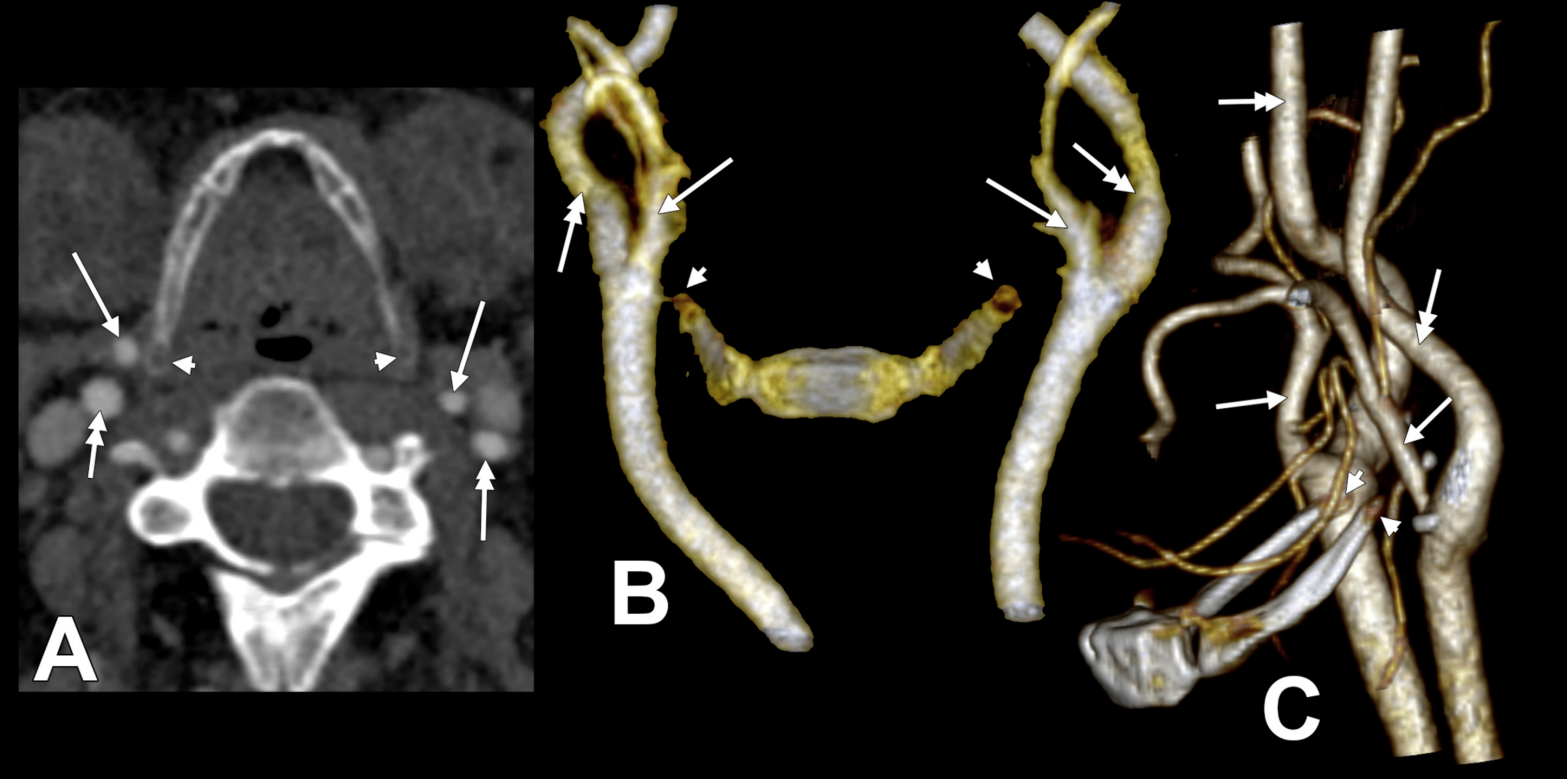
Bilateral ECA-GHHB combinations. A) Axial slice, inferior view, lateral (right)/postero-lateral (left); B) 3D volume rendering, anterior view, supero-lateral (right)/postero-superior (left); C) 3D rendering, left lateral view, supero-lateral (right)/posterior (left). Arrowhead denotes the hyoid tubercle; arrow denotes the ECA; double-headed arrow denotes the ICA. ECA: external carotid artery; GHHB: greater horn of the hyoid bone; ICA: internal carotid artery

**Figure 4:**
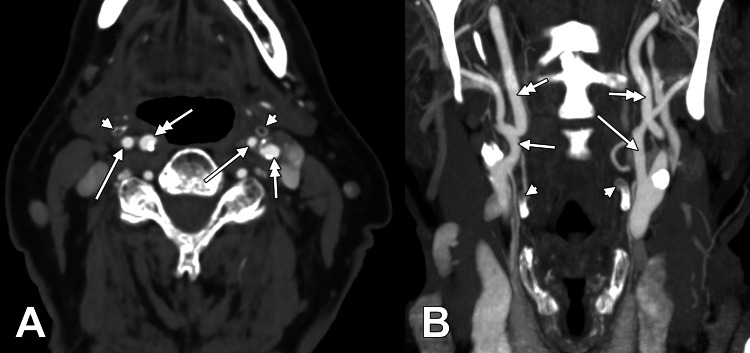
Bilateral ECA-GHHB anatomical variants. A) Axial slice, inferior view, medial/medial; B) Coronal slice, anterior view, superior (right)/supero-lateral (left). Arrowhead denotes the hyoid tubercle; arrow denotes the ECA; double-headed arrow denotes the ICA. ECA: external carotid artery; GHHB: greater horn of the hyoid bone; ICA: internal carotid artery

In total, 48 of 115 subjects (41.7%) showed bilateral symmetry and 67 of 115 (58.3%) asymmetry. Among symmetrical cases, lateral/lateral was most common (22 of 115; 19.1% of all subjects; 45.8% of symmetrical cases), followed by postero-lateral/postero-lateral (17 of 115; 14.8%; 35.4%) and postero-supero-lateral/postero-supero-lateral (4 of 115; 3.5%; 8.3%). Rarer symmetrical combinations included postero-medial/postero-medial (2 of 115; 1.7%) and medial/medial, posterior/posterior, and postero-superior/postero-superior (one case each; 0.9%). Figure 3 and Figure 4 illustrate representative bilateral combinations.

Tables 2-4 summarise bilateral combinations in males, females, and the whole cohort; Table 5 compares the most common patterns across groups; and Table 6 details symmetrical combinations by gender.

**Table 2:** Bilateral combinations of the external carotid arteries (ECAs) in males (N = 75). Data are presented as absolute counts (N) and percentages (%), calculated per subject. Statistical significance was defined as p < 0.05. Values are ranked from most common (postero-lateral/postero-lateral, 17.33%) to rarest (1.33% each).

Rank	Right Position	Left Position	Count	Percentage (%)
1	Postero-lateral	Postero-lateral	13	17.3
2	Lateral	Lateral	12	16.0
3	Lateral	Postero-lateral	11	14.7
4	Postero-lateral	Posterior	4	5.3
5	Lateral	Medial	3	4.0
6	Lateral	Supero-lateral	3	4.0
7	Postero-lateral	Lateral	3	4.0
8	Lateral	Postero-medial	2	2.7
9	Lateralised ECA	Posterior	2	2.7
10	Lateralised ECA	Postero-lateral	2	2.7
11	Postero-lateral	Supero-lateral	2	2.7
12	Postero-supero-lateral	Postero-supero-lateral	2	2.7
13	Supero-lateral	Posterior	2	2.7
14	Supero-lateral	Postero-supero-lateral	2	2.7
15	Lateral	Posterior	1	1.3
16	Lateral	Postero-supero-lateral	1	1.3
17	Lateralised ECA	Lateral	1	1.3
18	Medial	Medial	1	1.3
19	Postero-lateral	Postero-supero-lateral	1	1.3
20	Postero-medial	Postero-medial	1	1.3
21	Postero-superior	Postero-superior	1	1.3
22	Postero-supero-lateral	Postero-medial	1	1.3
23	Postero-supero-lateral	Postero-superior	1	1.3
24	Superior	Supero-lateral	1	1.3
25	Supero-lateral	Postero-lateral	1	1.3
26	Supero-lateral	Superior	1	1.3

**Table 3:** Bilateral combinations of the external carotid arteries (ECAs) in females (N = 40). Data are presented as absolute counts (N) and percentages (%), calculated per subject. Statistical significance was defined as p < 0.05. Values are ranked from most common (lateral/lateral, 25.0%) to rarest (2.5% each).

Rank	Right Position	Left Position	Count	Percentage (%)
1	Lateral	Lateral	10	25.0
2	Lateral	Postero-lateral	8	20.0
3	Postero-lateral	Postero-lateral	4	10.0
4	Postero-lateral	Posterior	3	7.5
5	Postero-lateral	Postero-supero-lateral	2	5.0
6	Postero-supero-lateral	Postero-supero-lateral	2	5.0
7	Lateral	Lateralised ECA	1	2.5
8	Lateral	Supero-lateral	1	2.5
9	Lateralised ECA	Postero-lateral	1	2.5
10	Lateralised ECA	Postero-supero-lateral	1	2.5
11	Posterior	Posterior	1	2.5
12	Postero-lateral	Lateral	1	2.5
13	Postero-lateral	Postero-superior	1	2.5
14	Postero-medial	Postero-medial	1	2.5
15	Postero-supero-lateral	Postero-lateral	1	2.5
16	Postero-supero-medial	Postero-superior	1	2.5
17	Supero-lateral	Postero-supero-lateral	1	2.5

**Table 4:** Bilateral combinations of the external carotid arteries (ECAs) in the overall cohort (N = 115). Data are presented as absolute counts (N) and percentages (%), calculated per subject. Statistical significance was defined as p < 0.05. The complete dataset is ranked by frequency.

Rank	Right Position	Left Position	Count	Percentage (%)
1	Lateral	Lateral	22	19.1
2	Lateral	Postero-lateral	19	16.5
3	Postero-lateral	Postero-lateral	17	14.8
4	Postero-lateral	Posterior	7	6.1
5	Lateral	Supero-lateral	4	3.5
6	Postero-lateral	Lateral	4	3.5
7	Postero-supero-lateral	Postero-supero-lateral	4	3.5
8	Lateral	Medial	3	2.6
9	Lateralised ECA	Postero-lateral	3	2.6
10	Postero-lateral	Postero-supero-lateral	3	2.6
11	Supero-lateral	Postero-supero-lateral	3	2.6
12	Lateral	Postero-medial	2	1.7
13	Lateralised ECA	Posterior	2	1.7
14	Postero-lateral	Supero-lateral	2	1.7
15	Postero-medial	Postero-medial	2	1.7
16	Supero-lateral	Posterior	2	1.7
17	Lateral	Lateralised ECA	1	0.9
18	Lateral	Posterior	1	0.9
19	Lateral	Postero-supero-lateral	1	0.9
20	Lateralised ECA	Lateral	1	0.9
21	Lateralised ECA	Postero-supero-lateral	1	0.9
22	Medial	Medial	1	0.9
23	Posterior	Posterior	1	0.9
24	Postero-lateral	Postero-superior	1	0.9
25	Postero-superior	Postero-superior	1	0.9
26	Postero-supero-lateral	Postero-lateral	1	0.9
27	Postero-supero-lateral	Postero-medial	1	0.9
28	Postero-supero-lateral	Postero-superior	1	0.9
29	Postero-supero-medial	Postero-superior	1	0.9
30	Superior	Supero-lateral	1	0.9
31	Supero-lateral	Postero-lateral	1	0.9
32	Supero-lateral	Superior	1	0.9

**Table 5:** Summary comparison by gender and in the overall cohort of the most common bilateral combinations (combos) of the external carotid artery position. Data are presented as absolute counts (N) and percentages (%), calculated per subject. Statistical significance was defined as p < 0.05. Gender differences were evaluated using Chi-square tests of independence (all χ² tests, p > 0.05).

Group	N	Unique Combos	Most Common Pattern	Most Common Count	Most Common Prevalence
Males	75	26	Postero-lateral/postero-lateral	13	17.3%
Females	40	17	Lateral/lateral	10	25.0%
Overall Cohort	115	32	Lateral/lateral	22	19.1%

**Table 6:** Symmetrical combinations of external carotid artery position types, by gender and in the overall cohort (combined). Data are presented as absolute counts (N) and percentages (%), calculated per subject. Statistical significance was defined as p < 0.05. Symmetry versus asymmetry was tested using a binomial test against an expected 50:50 distribution (z = 1.68, p = 0.093).

Position (Bilateral)	Males Count	Males %	Females Count	Females %	Combined Count	Combined %
Lateral	12	16.00%	10	25.00%	22	19.1%
Postero-lateral	13	17.33%	4	10.00%	17	14.8%
Postero-supero-lateral	2	2.67%	2	5.00%	4	3.5%
Postero-medial	1	1.33%	1	2.50%	2	1.7%
Medial	1	1.33%	0	0.00%	1	0.9%
Posterior	0	0.00%	1	2.50%	1	0.9%
Postero-superior	1	1.33%	0	0.00%	1	0.9%
Total	30	40.00%	18	45.00%	48	41.7%

Key statistical findings

Lateral ECA position was more frequent on the right than on the left (53/115, 46.1% vs 27/115, 23.5%; McNemar’s test, χ² = 20.86, p < 0.0001). ECA positions did not follow a uniform distribution (non-uniform positional distribution) (χ² = 389.157; p < 0.001). Lateral and postero-lateral positions together accounted for 155/230 sides (67.4%), significantly exceeding an expected 50% (p < 0.0001, binomial test). Dominance of lateral and postero-lateral positions was assessed using a binomial test (z = 5.22, p < 0.0001). Asymmetry was more frequent than symmetry (58.3% vs 41.7%; p = 0.093), indicating a non-significant trend toward asymmetry. No significant right/left difference for postero-lateral positioning (p = 0.349) was found. For the lateralised ECA, right-sided predominance (seven vs one left; p = 0.077) was observed but did not reach statistical significance due to the small sample size. No significant gender differences in ECA positioning were found (p > 0.05). Both sexes showed a right-sided lateral preference, with the postero-lateral position as the second most common. Lateralised ECA was rare in both sexes (<7% combined).

## Discussion

This CTA-based study defines an 11-type classification of ECA position relative to the GHHB and documents 32 bilateral combinations in 115 subjects. To our knowledge, this is the most detailed ECA-focused carotid-hyoid topography analysis to date [1-3,10].

From the present research, three main points emerge: (1) dominance of lateral/postero-lateral patterns; (2) marked right-sided lateral preference; and (3) frequent bilateral asymmetry. Lateral and postero-lateral positions comprised two-thirds of all ECAs. Textbook descriptions often imply a “typical” relationship but rarely quantify alternatives [1-4]. Our data suggest that lateral and postero-lateral patterns should be considered primary, with other positions as true variants. Nearly half of the right ECAs were lateral to the GHHB, whereas about one-quarter of the left ECAs were lateral to the GHHB. McNemar's test confirms that this is a systematic asymmetry rather than random variation. Asymmetry exceeded symmetry (58.3% vs. 41.7%), challenging the tacit assumption of bilateral vascular symmetry. Although this trend did not reach conventional statistical significance (p = 0.093), it has clear surgical relevance.

Developmental considerations

The ECA arises from the ventral pharyngeal artery and the ventral aortic system of the pharyngeal arches [19,20]. The hyoid bone derives mainly from the second and third arch cartilages, with contributions from the hypobranchial eminence [1,2,20,21]. Asymmetric growth, muscular development, and ossification may shift the relative positions of these derivatives. Our right-sided lateral preference may reflect consistent embryologic laterality patterns in the pharyngeal arch vasculature and hyoid complex [1,2,19-21].

Lateralised ECA variant

The lateralised ECA variant, with the ECA lateral or postero-lateral to the ICA, was seen in 3.5% of sides and was strongly right-sided. Reported prevalence ranges from 3% to 7%, typically with right-sided predominance, and is higher in older subjects [8,22-24]. Our data fit this range.

In this variant, branches may cross over the ICA, complicating vessel identification in carotid surgery [8,23-25]. It carries the risk of wrong-vessel ligation or injury during carotid endarterectomy, tumour resection, or neck dissection [8,24,25]; misinterpretation of angiography or Doppler studies [22,24]; and rare neurovascular compression, including hypoglossal nerve palsy, especially with high CB [15,24]. Hypoglossal palsy due to lateralised ECA and high CB has been reported, with tongue deviation, dysarthria, and lingual atrophy, and ECA ligation led to improvement [15].

Comparison with previous carotid-hyoid studies

Manta et al. reported variable carotid-hyoid topography and defined types that involve all carotid arteries, rather than ECA-specific patterns [1-3]. They reported the ECA lateral to the GHHB in 20.4% [2], comparable to our high lateral prevalence when bilateral data are considered. Karangeli et al. described 12 general carotid-hyoid types and found that “type 6” (ECA lateral to GHHB) was the most common (18.5%) [10]. Our more granular classification exposes complexity within such broad categories.

Existing work provides limited bilateral analysis and no detailed mapping of ECA-specific positional combinations [1-3,10]. Our 32 combinations, from common (lateral/lateral) to single-case rarities, offer a richer catalogue for surgical reference.

A comparative summary of major recent studies is given in Table 7, highlighting our ECA-specific focus, greater positional resolution, and structured bilateral analysis [1-3,10].

**Table 7:** Comparison with existing studies. Data are presented as descriptive categorical summaries. No inferential statistical testing was performed. ECA: external carotid artery

Study	N	Focus	ECA Positions	Bilateral Analysis	Gender
Present Study	115	ECA-specific positioning	11 detailed positions	32 combinations	Statistical testing
Manta et al. (2023) [2]	147	All carotid arteries	12 carotid types	Basic symmetry	No gender analysis
Karangeli et al. (2025) [10]	100	General carotid-hyoid	12 general types	Limited	Basic

Clinical implications: trauma, compression, and stroke

ECA-GHHB variants are usually silent but can become pathological when anatomy is perturbed. A case of ECA pseudoaneurysm followed an old hyoid fracture; the sharp fractured edge abutted the ECA, causing chronic injury and a 2.8-cm pseudoaneurysm that required surgical repair [18]. This illustrates how altered hyoid-artery contact can drive arterial wall damage and the formation of pseudoaneurysms. Plotkin et al. described recurrent stroke in a young woman with an elongated hyoid; the GHHB lay between the ICA (lateral) and ECA (medial), just above the CB [13]. During head rotation, the horn slid across the ICA, producing dynamic compression [13]. Similar reports show carotids moving into and out of retropharyngeal positions with swallowing and head movements, with repeated contact against the hyoid leading to endothelial injury, thrombus, and artery-to-artery embolism [14,16].

A GHHB projecting into a carotid artery can indent the vessel, rotating the ICA and ECA from their usual configuration [17]. Such cases reinforce the need to recognise variant carotid-hyoid relationships on imaging [9,11-17].

Surgical relevance

Our findings impact surgical approaches to the carotid triangle, neck dissection, and thyroid surgery [2,5,8-12,24,25]. On the right, surgeons should anticipate a high probability of lateral ECA relative to the GHHB (≈46%), which alters exposure and landmark relationships. On the left, ECA positions are more varied, with postero-lateral being the most frequent (35.7%). Because 58.3% of subjects display asymmetry, contralateral anatomy cannot be inferred from one side. This is relevant to staged bilateral procedures or to reoperations with scarring.

A high-lying hyoid may limit access to the CB, and an elongated GHHB can block exposure of the distal ICA. In some cases, partial hyoid resection may be needed [9,11-13]. GHHB proximity can complicate clamp placement, shorten arteriotomy, limit patch angioplasty, and restrict vessel mobilisation [9,11,12]. Postoperative issues may include compression, restenosis, or pseudoaneurysm formation [11,12,18].

ECA position may also affect the course of the superior thyroid artery (STA). Different GHHB-related STA courses (lateral, medial, and posterior to the horn) have been documented and influence the mobilisation of the thyroid superior pole [5]. Our data suggest that ECA and STA variants should be considered together when planning thyroid and upper neck surgery.

During neck dissection (levels II-III), variant ECA positions can shift lymph node fields relative to major vessels, changing the risk profile for vascular injury.

Gender and patient demographics

We found no significant sex differences in ECA position or bilateral patterns, consistent with previous work showing no considerable gender effects on ECA branching or CB level [26,27]. Nonetheless, females showed slightly higher right-sided lateral prevalence and symmetry, whereas males showed more left-sided postero-lateral dominance; these trends were not statistically significant.

Our male-predominant cohort reflects typical carotid imaging populations in stroke and cardiovascular disease (often 58%-75% male) [28]. Thus, our data are well aligned with the demographics of patients most likely to undergo carotid interventions.

Study limitations

This was a retrospective, single-centre CTA study. Functional correlations with symptoms or outcomes could not be systematically assessed. Patients with severe atherosclerosis (>50% stenosis) and those in whom vessel position might interact with plaque or stenosis progression were excluded.

Rare variants, particularly lateralised ECAs (eight sides), are associated with limited statistical power. Multi-centre, larger datasets are needed to refine prevalence estimates and explore associations.

Our sample was geographically and ethnically homogeneous, which may limit generalisability. Carotid and skeletal variations may show ethnic patterns [1-3,26,27]. Validation in diverse populations is warranted.

CTA is the current standard for carotid imaging but is susceptible to artefacts from positioning, contrast timing, and dental hardware [29,30]. We used standardised protocols and obtained high inter-observer agreement (κ = 0.82), suggesting that these factors did not significantly affect classification. An additional limitation is that CTA provides a static assessment of vascular-skeletal relationships. Dynamic changes in carotid-hyoid positioning may occur during physiological activities, such as swallowing, neck rotation, or phonation, which cannot be captured using static imaging. Consequently, potential functional or positional variability of ECA-hyoid relationships under dynamic conditions could not be evaluated in this study.

## Conclusions

ECA positions relative to the GHHB are highly variable but show clear patterns. Eleven distinct positional types and 32 bilateral combinations were identified. Lateral and postero-lateral ECA positions dominate and should be considered primary patterns. There is a marked right-sided preference for lateral ECA positioning. Bilateral asymmetry is more common than symmetry, limiting the predictive value of contralateral anatomy. Lateralised ECAs are uncommon but clinically essential and predominantly right-sided. These findings support the use of systematic, patient-specific imaging assessment before carotid, thyroid, and neck surgery. The proposed classification and prevalence data provide a practical framework for risk stratification, surgical planning, and anatomical teaching.

Future studies should validate this classification in larger, multi-centre, and multi-ethnic cohorts, incorporate dynamic imaging to assess positional variability, and explore correlations with surgical outcomes and procedure-specific complications.
